# Lichen cell factories: methods for the isolation of photobiont and mycobiont partners for defined pure and co-cultivation

**DOI:** 10.1186/s12934-022-01804-6

**Published:** 2022-05-09

**Authors:** Zakieh Zakeri, Stefan Junne, Fabia Jäger, Marcel Dostert, Volker Otte, Peter Neubauer

**Affiliations:** 1 Fachgebiet Bioverfahrenstechnik, Institut Für Biotechnologie, Technische Universität Berlin, Ackerstr. 76, 13355 Berlin, Germany; 2 Senckenberg Museum Für Naturkunde Görlitz, Am Museum 1, 02826 Görlitz, Germany

**Keywords:** *Ascomycota*, Lichen cultivation, Isolation method, Colony development, Pure culture, Axenic culture, Co-culture, Secondary metabolites, Biotechnology

## Abstract

**Background:**

Due to their huge biodiversity and the capability to produce a wide range of secondary metabolites, lichens have a great potential in biotechnological applications. They have, however, hardly been used as cell factories to date, as it is considered to be difficult and laborious to cultivate lichen partners in pure or co-culture in the laboratory. The various methods used to isolate lichen fungi, based on either the ascospores, the conidia, or the thallus, have so far not been compared or critically examined. Therefore, here we systematically investigate and compare the known methods and two new methods to identify the most suitable technology for isolation of fungi from lichens.

**Results:**

Within this study six lichen fungi species were isolated and propagated as pure cultures. All of them formed colonies within one month. In case of lichens with ascocarps the spore discharge was the most suitable method. Spores were already discharged within 2 days and germinated within only four days and the contamination rate was low. Otherwise, the soredia and thallus method without homogenization, as described in this work, are also well suited to obtain pure fungal cultures. For the isolation of algae, we were also successful with the thallus method without homogenization.

**Conclusion:**

With the methods described here and the proposed strategic approach, we believe that a large proportion of the lichen fungi can be cultivated within a reasonable time and effort. Based on this, methods of controlled cultivation and co-cultivation must now be developed in order to use the potential of lichens with regard to their secondary metabolites, but also for other applications.

**Supplementary Information:**

The online version contains supplementary material available at 10.1186/s12934-022-01804-6.

## Background

Lichens arise from a symbiotic relationship between a fungus, the mycobiont, and one or more phototrophic algae and/or cyanobacteria, the photobiont(s). With more than 30,000 species, lichens represent 20% of the currently known global fungal biodiversity; they even occur in the most extreme ecosystems on earth. Lichens produce a magnitude of natural compounds. From the 700 lichen-based substances whose structures have been clarified, many are exclusively found in lichens; they include depsides, depsidones, dibenzofurans, quinones, chromones, carotenoids, poly- and monosaccharides, aliphatic acids and others [1]. These so-called lichen compounds may provide more than 40% of the lichen’s dry weight [2] and are partly excreted as crystals on the surface of the fungal hyphae of the lichen in a natural growth environment [1].

Substances from lichens have been used from ancient times on, e.g. as dyes or for medical purposes, as pH indicator (Litmus), or as fragrances. Examples are *Evernia prunastri* (oakmoss) which is an important basic fragrance in the perfume industry. The toxic *Letharia vulpina* was historically used as a dye, but also to empoison wolves [3, 4]; yellow dye of *Xanthoria parietina* has been used for dyeing textiles [5]. Many of the lichen substances have been recently shown to have anticancer and antimicrobial activities and thus lichens could play an important role for the production of pharmaceuticals [6–8]. However, due to their slow growth and sensitivity to changing environmental conditions, collecting larger quantities of lichen for human use (e.g. Iceland moss *Cetraria islandica*, Reindeer moss *Cladonia* subgen. *Cladina*, beard lichen *Usnea*) in nature becomes more and more problematic not only from an economic point of view, but also in view of nature conservation. Collecting most of the traditionally used lichen species is prohibited by law in the countries of Central Europe [e.g. 9]. Development of standards forthe cultivation of relevant species and for biotechnological production of economically interesting lichen compounds can therefore considerably gain importance in the future as a continuous, steady supply of various natural materials.

Up to now, it has been extremely difficult to mimic the lichen symbiosis in the laboratory in large quantities of biomass, and subsequently the production of lichen substances. One reason is that contaminants in the lichen thallus like bacteria and parasitic fungi, among others, grow faster in a nutrient-rich laboratory medium than the lichen symbionts themselves. To obtain a pure lichen culture, the separation of the individual lichen partners is a prerequisite. The respective isolates can be incubated on solid or liquid media with various carbon and nitrogen sources [10, 11]. The optimization of environmental parameters such as light, pH-value, nutrient supply, humidity and temperature regimes are particularly relevant for the growth of lichens.

Culture conditions vary greatly between species and have to be identified specifically [11]. Most studies with isolated mycobionts have been conducted in order to examine either lichen re-synthesis and thallus development under laboratory conditions or the production of secondary metabolites [12]. Some of the lichen substances can be obtained with axenic (i.e. one-species, pure) cultures of lichenized fungi (lichen-associated fungi) under artificial stresses such as simulated day-night cycles, temperature fluctuations, strong light intensity or specific moisture regimes [13]. Most of the published studies were carried out, however, under laboratory conditions with the purpose to demonstrate the principle feasibility. We are not aware of any publication, in which such methods have been further developed from a process-related view, either to ensure mass production of lichens or a specific substance from them.

While the isolation of the lichen photobiont, i.e. the algae or cyanobacterium, is relatively straightforward due to the nutrient-poor media, which can be applied, the isolation of the mycobionts is still a lengthy trial-and-error process, often with high probability of a contamination, long incubation times and low success rates. In our opinion, for a wider use of lichens as cell factories, it is important to critically evaluate and further improve the current isolation techniques and strategies, as well as the culture media used for isolation of mycobionts.

The studies about the isolation of lichen partners used either lichen thallus fragments or ascospores. The isolation of lichen thallus fragments was originally described by Yamamoto et al. [14] and subsequently used and modified by other authors [10, 15–18]. The isolation from spores was described by Ahmadijan [19] and Yoshimura et al. [20]. Recently Černajová and Škaloud [21] used soredia, and Zakeri et al. [22] used soredia and thallus without washing and homogenization to isolate lichen partners. There are some successful isolations described for lichen fungi from different groups, e. g. foliose, crustose and fruticose lichens [e.g. 23–25]. The success of different methods for a specific species, however, was not compared yet. After obtaining axenic (i.e. pure) cultures of the lichen partners, isolates are normally cultivated on various solid and soft agar media with different nutrients, C- and N-source concentrations [10, 15, 16, 26]. Lichen symbionts grow much faster as cell aggregates in lab conditions than in nature, e.g. 6 g dry biomass of *U. ghattensis* on Petri dishes were achieved within 2 months [27]. Nevertheless, growth is still slower than that of many other microorganisms. To our knowledge, there are still no real systematic optimization studies that would be the basis for the development of industrial applications.

In order to facilitate access to the biotechnological exploitation of lichens, the aim of this current study was to describe the application and comparison of different methods for the isolation of the individual lichen symbionts. These methods comprise of the use of the special sexual and asexual structures in lichens (apothecia, pycnidia, thallus-fragment) and enable a cultivation of the mycobiont and photobiont of lichens in pure cultures. Our goal was to propose a simple, efficient and strategic approach for the selective isolation of lichen partners and their better growth in the biotech lab.

## Results

In this study, we compared five different methods for isolating fungi from lichens in combination with one method for algae isolation. Six lichens were selected for the method comparison (Table 1). All lichen-associated fungi and four lichen-associated algae from six lichens were isolated.

**Table 1:** Isolation of lichen taxa with the different methods

Taxon/isolation method	Isolation of fungi					Isolation of Algae
	Ascospores	Conidia	Soredia	Yamamoto-Thallus	Thallus	
*Cladonia macilenta*	−	−	+	−	n. a.^a^	+
*Cladonia fimbriata*	−	−	+	−	n. a.^a^	+
*Protoparmeliopsis muralis*	+	−	n. a.^b^	−	+	−
*Parmelia sulcata*	n. a.^c^	−	+	−	+	−
*Circinaria contorta*	+	−	n. a.^b^	−	+	+
*Xanthoria parietina*	+	−	n. a.^b^	−	+	+

+ positive result, − no result, n.a. method was not applied

^a^The method was not applied as it was difficult to find a soredia-free parts on the thallus of this species

^b^The method was not applied as the species usually does not develop apothecia

^c^The method was not applied as the species does not have soredia

Table 1 summarizes the isolation results of lichen partners with the different methods.

**Fig. 1:**
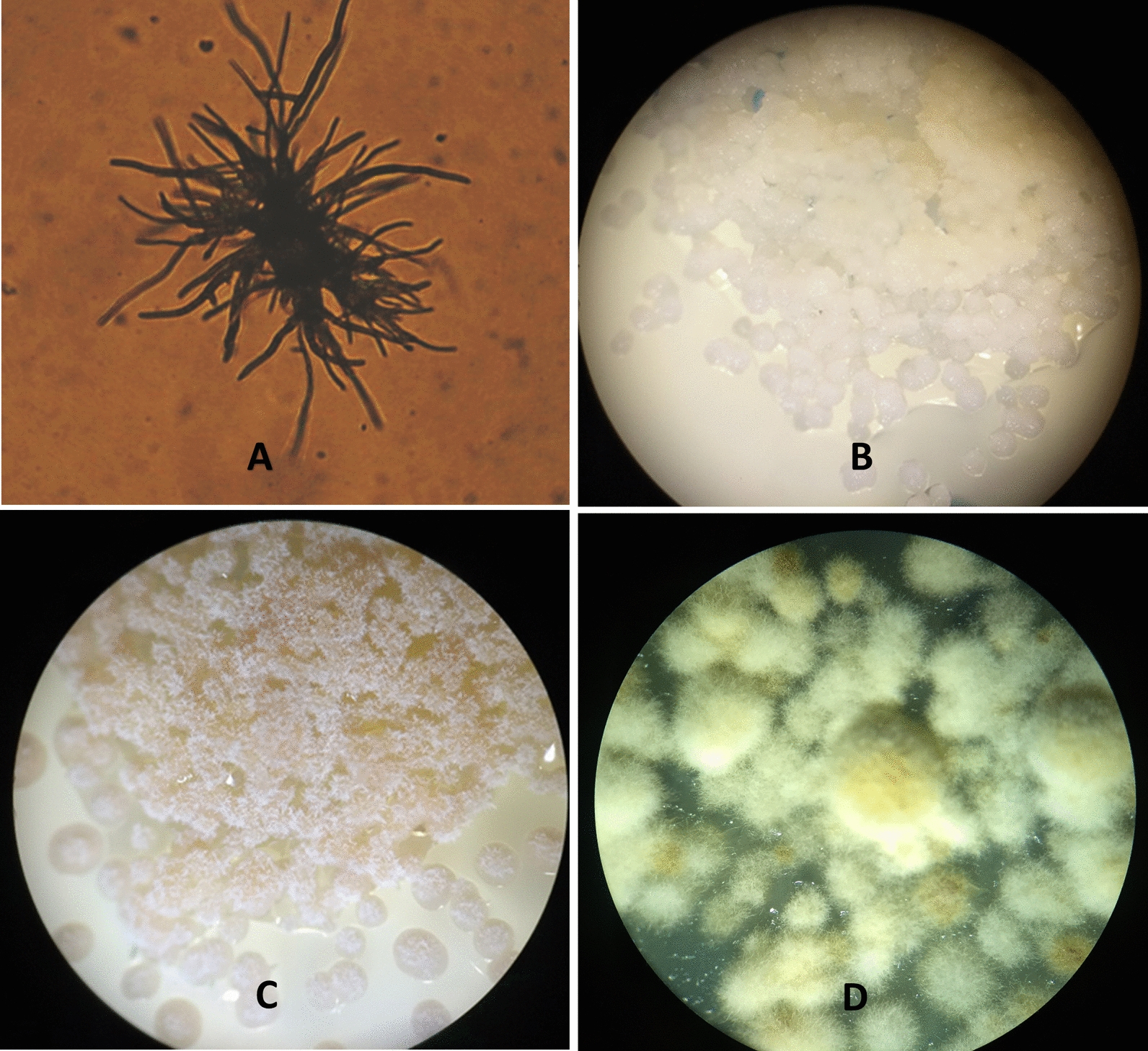
Ascospore discharge method—**A** Germinated ascospores of *Protoparmeliopsis muralis* after 6 days (200 × magnification); Colony formation after 75 days of **B** *Circinaria contorta* (20 × magnification); **C** *Xanthoria parietina* (20 × magnification) and **D** *P. muralis* (40 × magnification). **A** and **C** from Zakeri et al. [22]

The most successful method in this study was the isolation from ascospores. When this method was applied, the ascospores of three out of five species discharged after 2 days at 22 °C in the dark. All the samples with discharged spores germinated after 4 days; all of them built colonies after 30 days (Fig. 1). The contamination rate of 15% was much lower in this than at all other methods (more details in the discussion). The results from all taxa grown in different media and treated with this method are summarized in Table 2.

**Table 2:** Result of the ascospore discharge method when different media were applied

Medium / Taxon	Ascospores discharged	Germination	Colony development
MBB			
* Cladonia macilenta*	−	−	−
* Cladonia fimbriata*	−	−	−
* Protoparmeliopsis muralis*	+	+	+++
* Circinaria contorta*	+	+	+++
* Xanthoria parietina*	+	+	+++
LB			
* Cladonia macilenta*	−	−	−
* Cladonia fimbriata*	−	−	−
* Protoparmeliopsis muralis*	+	+	++
* Circinaria contorta*	+	+	++
* Xanthoria parietina*	+	+	++
WA			
* Cladonia macilenta*	−	−	−
* Cladonia fimbriata*	−	−	−
* Protoparmeliopsis muralis*	+	+	+
* Circinaria contorta*	+	+	+
* Xanthoria parietina*	+	+	+
MY			
* Cladonia macilenta*	−	−	−
* Cladonia fimbriata*	−	−	−
* Protoparmeliopsis muralis*	+	−	−
* Circinaria contorta*	+	−	−
* Xanthoria parietina*	+	−	−

+ : positive result; −: no result; +, ++, +++ represent the speed of growth of the colonies

**Fig. 2:**
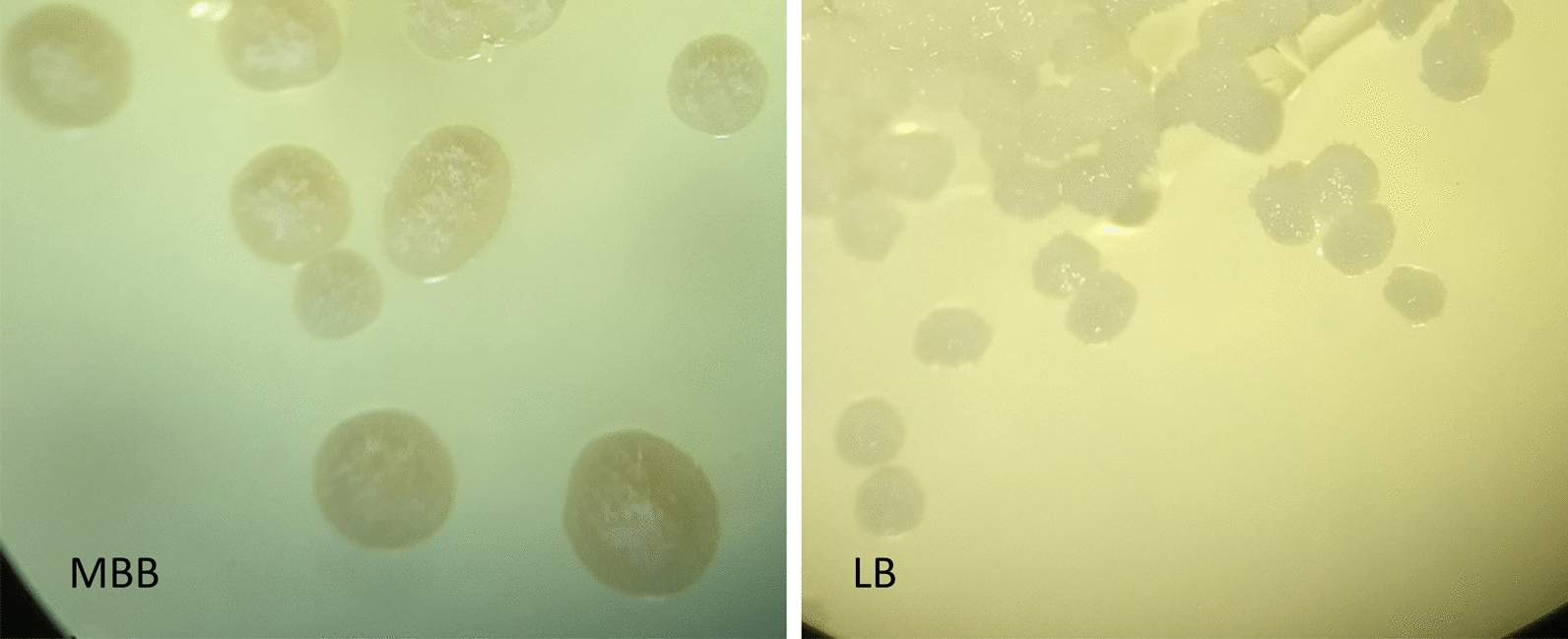
Ascospore discharge method—Colony formation of ascospores (*X. parietina*) in MBB and LB medium after 1 month (20 × magnification)

The successful isolation and growth of different fungi by the ascospore discharge method was correlated to the type of medium. The different media contained different inorganic and organic compounds, they were differently enriched by nutrients, such as sugars, metal compounds, amino acids and vitamins. The spores germinated on all media except for the MY medium, where we did not observe any germination of spores, as described before by Yoshimura et al. [20]. The germinated spores grew faster in MBB medium than in LB, and, as expected, much slower in WA media (Fig. 2). Thus, we decided to use MBB and BBM for all other methods. We also preferred BBM because it showed much less contamination.

We did not get any isolation by the Yamamoto-thallus method for the lichen forming fungi, but the method was successfully applied for the isolation of algae from *X. parietina* in one inoculate. The isolation success rate was below 1%; most probably due to the washing process, no growth of lichenized fungi was observable, although growth of contaminants were obtained. The conidia method was not successfully applied as no conidia were discharged from pycnidia (asexual fruiting bodies).

**Fig. 3:**
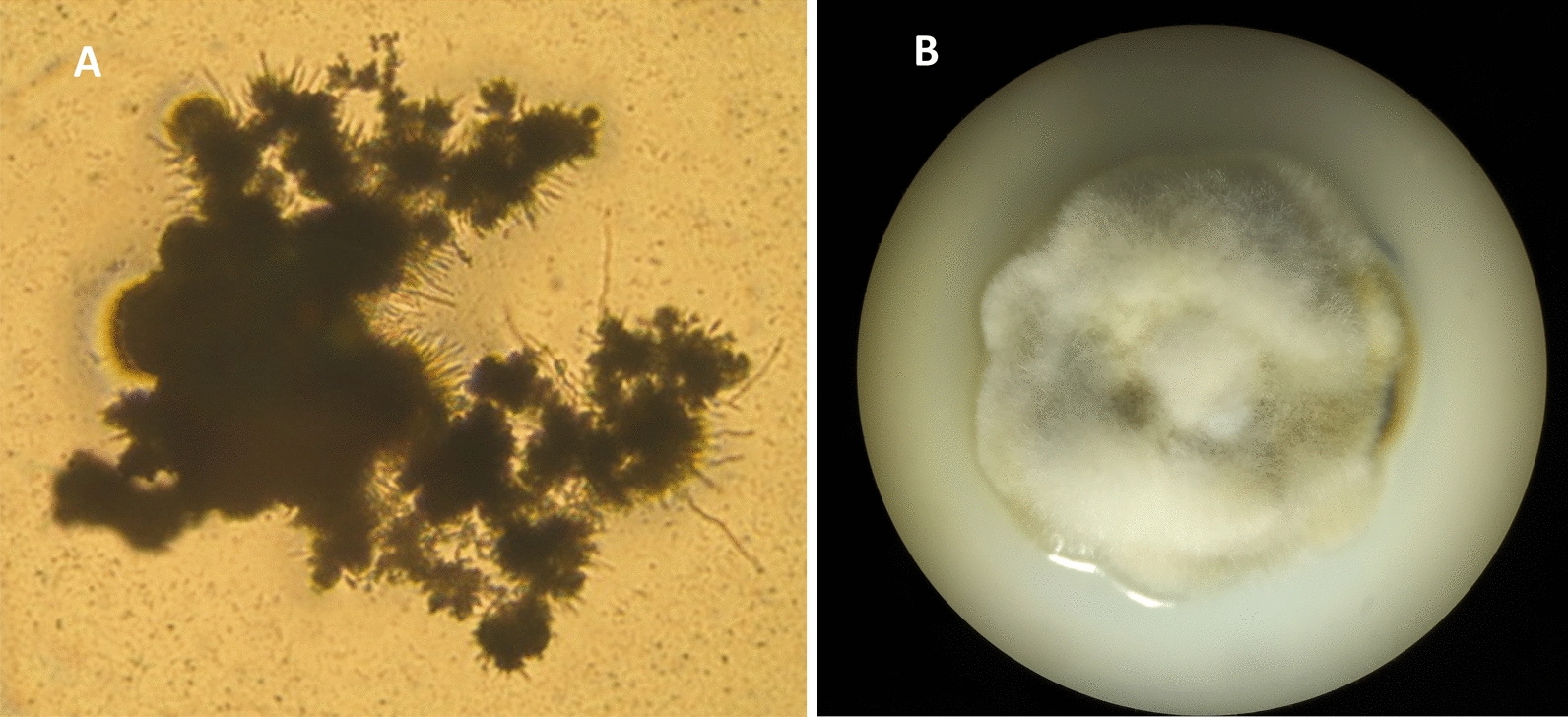
Isolation of lichen forming fungi from soredia—Isolated soredia from *C. fimbriata*; **A** after one week of cultivation (100 × magnification); **B** after 2 months of cultivation (20 × magnification). From Zakeri et al. [22]

**Fig. 4:**
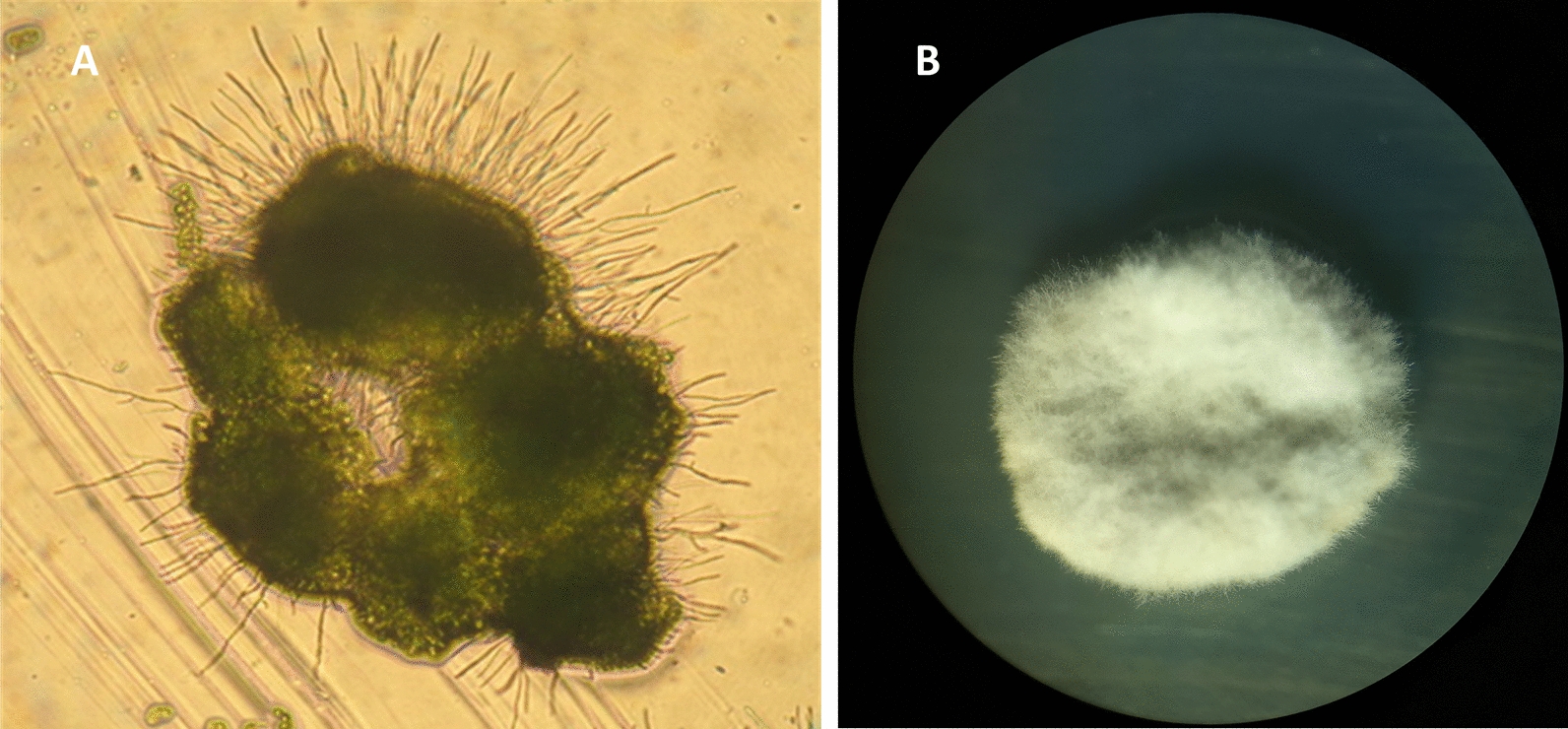
Isolation and cultivation of lichen forming fungi from thallus fragments—An isolated thallus part from *X. parietina*; **A** Colony after one week of cultivation (200 × magnification) with fungal mycelium and algae; **B** Colony of fungal mycelium after 2 months of subcultivation (20 × magnification). From Zakeri et al. [22]. For details see section “Subcultivation of the mycobiont in the Materials and Methods section below

The other methods described in this study, the isolation from soredia and thallus fragments, respectively, showed good success rates of 59% (56 successful isolates of 96 inoculates, for an example see Fig. 3) and 17% (22 successful isolates of 128 inoculates, for an example see Fig. 4). The soredia method for *L. muralis*, *C. contorta* and *X. parietina* was not applied as these species do not have soredia. The thallus fragment method was not applied for *C. macilenta* and *C. fimbriata*, as good results were obtained with the soredia method and it was difficult to find a soredia-free part on the thallus of this species.

The calculated success rates for the various methods are shown in Table 3 for BBM and in Table 4 for MBB medium.

**Table 3:** The success rate of isolation methods in BBM medium

Method	Inoculates	Successful isolations	Success rate [%]
Ascospore	44	28^a^	63.63%
Thallus Yamamoto	202	1	0.49%
Thallus (this study)	64	10	15.6%
Soredia	48	31	64.5%
Conidia	28	no conidia discharged	–
Algae	152	10	%

^a^Ascospores were only discharged in 3 species, which lowered the success rate

**Table 4:** The success rate of isolation methods in MBB medium

Method	Inoculates	Successful isolations	Success rate [%]
Ascospore	46	30^a^	65.21%
Thallus Yamamoto	210	0	0.0%
Thallus (this study)	64	12	18.75%
Soredia	48	25	52%
Conidia	26	no conidia discharged	–
Algae	151	7	4.6%

^a^Ascospores were only discharged in 3 species, which lowered the success rate

The contamination rates were high in the Yamamoto (approx. 80%) and in the soredia and thallus-based methods (both approx. 50%) and significantly lower in the conidia (approx. 20%) and in the ascospore discharge (approx. 15%) methods.

DNA sequencing was performed for taxonomic identification of the isolated cultures for future studies on the interaction of defined lichen co-cultures and for a further provision of data about the detailed conditions in the isolation studies. The GenBank-Number of the ITS marker from cultivated samples is shown in Table 5.

**Table 5:** The GenBank-Number of the ITS marker from isolated lichen partners in subculture

Species	GenBank-Number of the Isolated fungi	Culture numbers	Species	GenBank-Number of the Isolated algae	Culture numbers
*Cladonia macilenta*	OK491796	Zakeri F-0001	*Asterochloris italiana*	OK491797	Zakeri A-0001
*Cladonia fimbriata*	OK491791	Zakeri F-0002	*Asterochloris lobophora*	OK491800	Zakeri A-0002
*Protoparmeliopsis muralis*	OK491795	Zakeri F-0003	No result^a^		
*Parmelia sulcata*	OK491792	Zakeri F-0004	*Coccomyxa sp.*^b^	OK491799	Zakeri A-0004
*Circinaria contorta*	OK491794	Zakeri F-0005	*Trebouxia sp*.	OK491801	Zakeri A-0005
*Xanthoria parietina*	OK491792	Zakeri F-0006	*Trebouxia decolorans*	OK491798	Zakeri A-0006

^a^No algae were isolated

^b^No lichen-associated algae were isolated

## Discussion

**Fig. 5:**
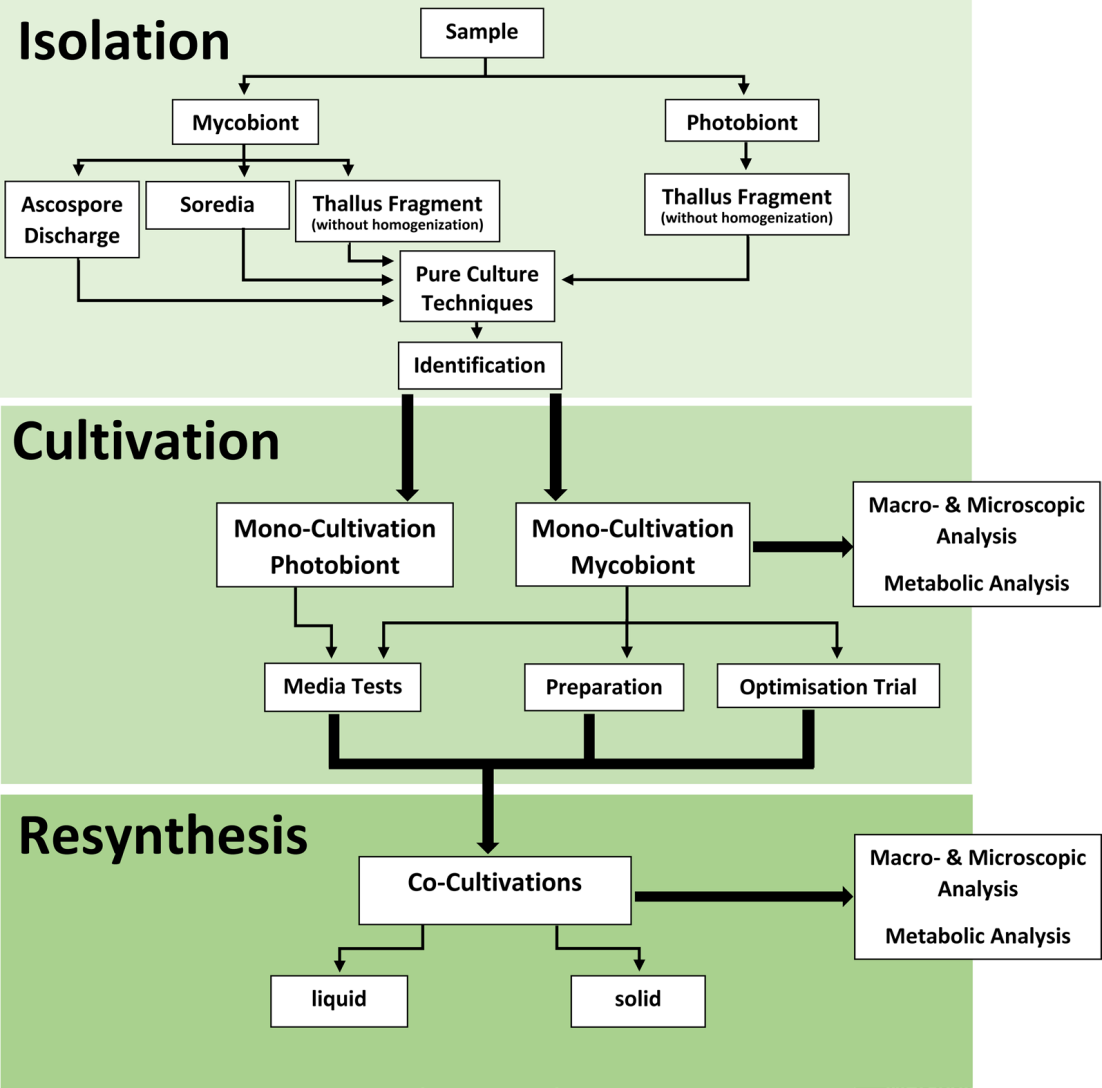
Proposed workflow and steps for a biotechnological valorization of lichens

The present study aimed to compare and finally identify the most suitable methods for the isolation of lichen partners by using a range of methods and media combinations. The resulting workflow proposed in Fig. 5 shall provide reliable results for the further investigation of the growth characteristics and the product spectrum of the isolates and their use in defined co-cultures to resynthesize lichen under controllable conditions. A better understanding of the individual strains supports the optimization of growth conditions, which might help to increase biomass formation and finally improve side product identification. The results as obtained in this study clearly showed the advantages, disadvantages and remaining challenges for the application of the different methods for the isolation of either fungi or algae out of the lichen consortia in order to obtain pure axenic cultures within few weeks.

The most common method used to isolate lichen partners from thalli is to homogenize lichen thalli with some additional flow steps [23, 28, 29]. Recently, Černajová & Škaloud [21] used soredia without washing and homogenization to isolate lichen partners. In their method, they picked the entire soredium directly from the thalli with a sterile needle and placed it onto cultivation media to isolate the lichen partners (algae and fungi) and other fungi co-dispersed with soredia. In contrast, we took the soredia and placed them in a sterile Petri dish for the examination under the stereomicroscope. The smaller photobiont-free fragments were then picked out and transferred to agar media with sterile wood sticks to obtain pure fungal cultures. In the thallus method without homogenization we tried to select photobiont-free fragments to obtain pure fungal culture and mycobiont-free fragments to obtain pure algal cultures.

In our actual study, algae from lichen species were isolated successfully with the thallus method introduced in this work (see Methods section). The algae were isolated on BBM medium, which contains no sugar and thus prevents fungal contamination. The algae on the Petri dishes were visible within a month and were mostly grown in between fungal mycelia. Few repeated subcultures of the algae in a new Petri dish were required to obtain axenic cultures.

Results showed that the ascospore discharge is a very suitable method, which is easy to handle and only rarely leads to contamination. According to Ahmadijan [12], the time, after which the first spores discharge in the Petri dish appears, varies widely and depends on the species as well as on the condition of the apothecia at the time of collection, treatment of the thalli after collection and the age of the individual ascocarps. Also in the experiment by Sangvichien et al. [24] the apparent seasonal effect on ascospore discharge and germination was examined and was found to be significant in some species. The samples used in our study were collected in spring (April) and treated freshly. Here, the ascospore discharge was observed within two and four days after placing an apothecium in the Petri dish.

Aside from these advantages, the ascospore discharge has also some disadvantages, e.g. apothecia may not discharge spores, spores do not always germinate in vitro, and not all of the germinated spores form colonies. Ascospore discharge, however, is seen as the method of choice for the lichen species with ascomata, as it was successfully applied in most cases in this study and the rate of contamination was very low. In cases, in which this method does not lead to a desirable result, we recommend the isolation of soredia, if present, or as an alternative the thallus method without homogenization. In our study however, the thallus method as described by Yamamoto was not effective, neither for the fungi nor for the algae. One likely reason is that the samples exhibited a high sensitivity against the ethanol which is used for decontamination. Also, other washing methods did not exhibit better results. It seems that these methods with ethanol and Tween are useful for the isolation of the lichenicolous fungi, i.e. non-lichen-forming fungi which live on and in a lichen and also for the expanded microbial community [17, 30], but cannot be recommended for the isolation of the original lichen symbionts.

In the present study, the thallus method according to Yamamoto had a much higher contamination rate than any of the other methods (about 80%). In case of the soredia and thallus-based methods, the contamination rate was about 50%; it was always necessary to cut out the inoculated pieces without contamination and re-transplant them into new Petri dishes in order to prevent overgrowth. Another reason for the poor results might be the homogenization of the samples, which increases the possibility of contaminations and hinders the growth of the inoculate among the other parasitic or surface-grown microbes and fungi on plates. The contamination rate in the ascospore discharge method was only about 15%, and thus much lower than for the other methods, which makes this method especially suitable. Another advantage of the ascospore discharge method is that more biomass is produced compared to other methods, due to the large number of spores which are normally discharged on a plate.

The result of the ascospore discharge showed that MBB medium yielded the best results for cultivating spores and the subsequent formation of colonies compared to all other media (Table 2). In general, MBB proved to be the most suitable medium for the isolation of the tested lichen taxa, as all the isolates (lichen mycobionts and algae) grew faster on it than on any of the other media (Fig. 2). Verma and Behera [27] showed, however, that the lichen species in their experiments grow at different rates and that each of them requires a specific medium. Thus it might be recommended to test different media if MBB is not successful.

The temperature has a very strong influence on the isolation success. This study showed that a temperature between 16 and 20 °C provides better conditions for isolation and cultivation of the samples than a higher temperature between 24 and 30 °C or lower temperatures between 12 and 16 °C. While hardly any positive results were gained at 30 °C, many samples performed well at 18 °C. Also, in the study by Bando and Sugino [31], lichen thalli from *Parmotrema tinctorum* collected from Japan grew faster at 20 °C than at 10 °C and 30 °C. In another study, Yoshimura et al. [32] were able to induce cultivated cell aggregates from some Antarctic lichens that had been stored in the refrigerator for several years. In addition, the mycobiont of *Umbilicaria aprina* from the Antarctic was strongly adapted to the cold; it grew faster at a very low temperature (5 °C) than at a moderate temperature (15 °C). The mycobiont *Umbilicaria decussata*, found in the antartic, grew, however, as good at moderate temperatures, similar to *Umbilicaria muhlenbergii* and *Lasallia pensylvanica*. More studies are required to understand the potential adaptation mechanisms and the role of temperature for the interaction between fungi and algae in order to explain the different temperature sensitivities among lichens.

## Conclusions

In summary, a workflow is proposed as shown in Fig. 5. It consists of three major steps: (i) isolation, which includes the sampling and the isolation of symbionts, the purification and the identification of the isolates; (ii) the mono-cultivation, which contains the optimization of the growth conditions of both, the photobiont and the mycobiont; and (iii) the resynthesis, attempted with co-cultivations between a photobiont and a mycobiont on solid or in liquid media.

Step one of the proposed workflow in Fig. 5 as a prerequisite for steps two and three enables the application of bioprocess development from small, parallel cultivation up to a larger scale with monitoring and control of the co-culture. Although not yet widely applied, many novel tools and methods do exist or are currently in development to achieve stable co-cultures for bioproduction purposes. These comprise of visualization methods of individual cells in an automated manor like in-line microscopy, particle shape measurements or pigment quantification, among others [33], which became applicable in defined co-cultures. Since the ascospore discharge method and isolation from soredia and thallus were very effective and led to axenic cultures unlike other methods used so far, they represent an important basis for further investigation of and bioprocess development with lichens and the valorization of their manifold products.

With the result of the present study, we were able to isolate and cultivate more than 20 lichens and were able to identify the lichen substances in the cultivation with TLC and HPLC methods. The results of these experiments will be processed and published subsequently.

## Materials and methods

### Sampling

Six lichen species, five with ascocarp, were collected in Berlin (Grunewald) in April 2020: *Cladonia macilenta* Hoffm.*, Cladonia fimbriata* (L.) Fr.*, Protoparmeliopsis muralis* (Schreb.) M. Choisy*, Parmelia sulcata* Taylor*, Circinaria contorta* (Hoffm.) A. Nordin, Savić & Tibell and *Xanthoria parietina* (L.) Th. Fr. All the collected lichens are associated with green algae. The materials were used freshly or stored in the freezer for later investigations. Before the frozen material was used, it was equilibrated for a few hours.

### Isolation of lichen forming fungi from lichen

Five different methods were used for the isolation of the mycobiont from lichens:

1. **Ascospore discharge** (use of sexual spores in apothecium): This method is described by various authors, but the basic principle is the same [2, 12]: The surface of the specimen was cleaned with a brush to remove any remaining soil and debris. A sterile scalpel was used to dissect specimen to obtain small portions with ascomata. These were then attached with scotch tape to the inside of the lid of a 5 cm diameter Petri dish filled with agar under a clean-bench. The Petri dish was placed upside down so that the spores are shot upwards onto the overlying layer of Agar with different media (WA, MYE, LB and MBB). The Petri dishes were stored in the dark at 22, 14 and 28 °C (± 2 °C). The agar surface was examined every second day with a stereo binocular microscope. Once ascospores had been trapped, the upper lid was exchanged for a new clean one. Germination and growth were both observed every 2—3 days under the light microscope and contaminations were cut out.
2. **Conidia discharge** (asexual spores produced in pycnidia)**:** the use of conidia to isolate lichen-fungi were previously used by Vobis G. [34] and Crittenden P. D. [23]. In this study the pycnidia were processed in the same way as described above for the ascospore discharge method.
3. **Isolation from soredia** (asexual reproductive organ): This method is introduced in Zakeri et al. [22]. A small part of the thalli with soredia and without visible contamination and parasites were selected under the stereomicroscope. Soredia that were visibly free of parasites and contamination were picked with a sterile wood stick directly from the thallus under a stereomicroscope and put into a sterile Petri dish. The soredia were examined under the stereomicroscope, the smaller photobiont-free fragments were picked and transferred to agar media with sterile wood sticks (each Petri dish with 8 tiny pieces / inocula).
4. **Isolation from thallus fragments** (the entire body of lichens): This method is introduced in Zakeri et al. [22]. A small part from thallus, free of contamination and parasites, was selected. The surface of the thallus was carefully scratched with sterile tweezers into a sterile Petri dish. The small, scratched thallus part was examined under the stereomicroscope, the smaller photobiont-free fragments were picked and transferred to agar media with sterile wood sticks (each Petri dish with 8 tiny pieces / inocula).
5. **Yamamoto-thallus methods**: this method was firstly described by Yamamoto et al. [14]. We applied the method with two additional washing protocols (a and b): small pieces of thallus were cut from apical regions, washed twice for 15 min with distilled and sterilized water, washed with:
6. Ethanol: 10 s with 70% ethanol, 10 s with 2% NaOCl
7. Tween: 30 min with 500 µL Tween 80 (diluted 1:10)
8. And finally, twice for 5 min with sterile water.

The pieces were mixed in a 3—5 ml sterile water using a mortar and pestle. The resultant suspension was sieved through 500 µm and 150 µm nylon mesh filters. The small pieces of thallus retained on the 150 µm sieve was examined under a stereomicroscope and the smaller photobiont free fragments were picked out and transferred to agar media using sterile wood sticks (each Petri dish with 8 tiny pieces / inocula).

### Growth media

The following nutrient media were used: Water-Agar (WA; [35]); Malt-Yeast Extract (MYE; [12]); Lilly Barnett (LB; [36]); modified Bold’s Basal medium (MBB; [22]), adjusted to pH-values between 6 and 7.

For the ascospore discharge method, four Petri dishes of each medium were inoculated for each species, resulting in a total of 16 Petri dishes (4 Petri dishes with 4 different media) and 35 inocula for each species [16 plates × 2 or 3 ascocarps per plate) and a total of 178 (≈ 35 × 5) inocula for 5 from 6 species, which were applied in this method (*Parmelia sulcata* do not have apothecia).

For other methods (conidia, soredia, thallus and Yamamoto), only MBB and Bold’s basal medium were used (BBM; [37]). Two Petri dishes of each medium were inoculated with each species, which resulted in 10 inocula for each species when following the protocol of the conidia method (2 Petri dishes × 2–3 pycnidia per plate × 2 media) and 32 inocula per species when following the protocols of the soredia, thallus and Yamamoto method (2 Petri dishes × 8 inocula × 2 media). For the Yamamoto method, we used two different washing protocols that increased the number of inocula in this method (2 Petri dishes × 8 inocula × 2 media × 2 washing steps).

### Subcultivation of the mycobiont

All Petri dishes were examined 3 times a week in order to prevent contaminations as quickly as possible, by removing the well-colonized parts of the plates without contamination on a new agar plate with MBB. The Petri dishes of the fungi isolate were incubated at 16–20 °C with alternating photoperiods of 8 h light and 16 h dark. The fungal colonies were mostly free of algal cells, but in a few cases where algal cells were present, the plate was kept in the dark for 1 week or the subcultures were provided to obtain axenic fungal cultures.

### Isolation of algae from lichen

The same procedure for isolating the mycobiont from the lichen thallus (our method described in the methods section) was used to isolate algae (mycobiont-free fragments) from the scratched thallus plate. The isolation was performed with BBM (Bold’s Basal Medium), which contains no sugar and thus prevents fungal contamination. The algae were cultivated with alternating light–dark phases (10 h with light, 14 h within the dark) at 20–22 °C. The plates were investigated 3 times a week. For each lichen species, 4 Petri dishes were then inoculated with algae isolate (each Petri dish with 8 tiny pieces / inocula). After repeated subculture of the algae in a new Petri dish, axenic cultures were obtained.

### Identification of the isolated fungi and algae

DNA analyses were performed to confirm the identities of the fungal and algal isolates. DNA was extracted from the fresh lichen material and the isolated fungal and algae subcultures from it, which were dried and grinded with a mill MM301 (Retsch GmbH, Germany). The grinded material was processed as described in Zakeri et al. [38, 39].

The primer pairs ITS1F [40] and ITS4 [41], ITS1LM [42] and ITS2KL [43] were used for the PCR amplifications of the ITS region of the mycobionts. The primer pairs ITS1T and ITS4T [44], nr-LSU-0012–3’ Algal and nr-SSU-1780–5’ Algal [45] were used for the PCR amplifications of the ITS region in algae. PCR amplifications were performed in a volume of 12.5 μL containing 2 μL undiluted DNA, 0.5 μL of each primer (10 nM), 6.4 μL of sterile water, 1 μL dNTP (2 nM), 1 μL s–buffer, 1 μL MgCl_2_, 0.1 μL Taq–polymerase (Peqlab). Thermal cycling parameters were as follows: initial denaturation for 5 min at 95 °C, followed by 30 cycles of 1 min at 95 °C, 1 min at 53 °C, and 1 min at 72 °C. Following the last cycle, a final extension for 5 min at 72 °C was executed. The amplification products were examined by electrophoresis on 1% agarose gels with ethidium bromide staining. Both DNA strands of the PCR product were sequenced on an ABI 3730 by LGC Genomics GmbH.

## Supplementary Information

**Additional file 1: Table S1.** Individual cultures and sample numbers present in the study.

## Data Availability

Individual cultures and specimen numbers are given in Additional file 1: Table S1.

## References

[1] Huneck S, Yoshimura I (1996). Identification of Lichen Substances.

[2] Masuch G (1993). Biologie der Flechten.

[3] Moberg R, Holmåsen I. Flechten von Nord- und Mitteleuropa. Ein Bestimmungsbuch, Gustav Fischer Verlag: Stuttgart, Jena, New York. 1992. ISBN 13: 9783437204715

[4] Schade A (1954). Über Letharia vulpina (L.) Vain. und ihre Vorkommen in der Alten Welt. Ber Bayer Bot Gesell..

[5] Shukla P, Upreti DK, Upreti D, Divakar P, Shukla V, Bajpai R (2015). Lichen dyes: current scenario and future prospects. Recent advances in lichenology.

[6] Basile A, Rigano D, Loppi S, Di Santi A, Nebbioso A, Sorbo S, Conte B, Paoli L, De Ruberto F, Molinari AM, Altucci L, Bontempo P (2015). Antiproliferative, antibacterial and antifungal activity of the lichen xanthoria parietina and its secondary metabolite parietin. Int J Mol Sci.

[7] Molnár K, Farkas E (2010). Current results on biological activities of lichen secondary metabolites: a review. Z Naturforsch C J Biosci.

[8] Brunauer GC, Stocker-Wörgötter E (2005). Culture of lichen fungi for future production of biologically active compounds. Symbiosis.

[9] Bundeartenschutzverordnung. Verordnung zum Schutz wild lebender Tier- und Pflanzenarten (Bundesartenschutzverordnung—BArtSchV). 2005. https://www.gesetze-im-internet.de/bartschv_2005/BJNR025810005.html

[10] Behera BC, Mahadik N, Morey M (2012). Antioxidative and cardiovascular-protective activities of metabolite usnic acid and psoromic acid produced by lichen species Usnea complanata under submerged fermentation. Pharm Biol.

[11] McDonald TR, Gaya E, Lutzoni F (2013). Twenty-five cultures of lichenizing fungi available for experimental studies on symbiotic systems. Symbiosis.

[12] Ahmadjian V (1993). The lichen symbiosis.

[13] Stocker-Wörgötter E (2008). Metabolic diversity of lichen-forming ascomycetous fungi: culturing, polyketide and shikimate metabolite production, and PKS genes. Nat Prod Rep.

[14] Yamamoto Y, Mizuguchi R, Yamada Y (1985). Tissue cultures of Usnea rubescens and Ramalina yasudae and production of usnic acid in their cultures. Agric Biol Chem.

[15] Behera BC, Verma N, Sonone A, Makhija U (2009). Optimization of culture conditions for lichen Usnea ghattensis G. Awasthi to increase biomass and antioxidant metabolite production. Food Technol Biotechnol.

[16] Savale SA, Pol CS, Khare R, Verma N, Gaikwad S, Mandal B, Behera BC (2016). Radical scavenging, prolyl endopeptidase inhibitory, and antimicrobial potential of a cultured Himalayan lichen Cetrelia olivetorum. Pharm Biol.

[17] Muggia L, Kopun T, Grube M (2017). Effects of growth media on the diversity of culturable fungi from lichens. Molecules.

[18] Guzow-krzemińska B, Stocker-Wörgötter E (2013). In vitro culturing and resynthesis of the mycobiont Protoparmeliopsis muralis with algal bionts. Lichenologist.

[19] Ahmadjian V (1961). Studies on lichenized fungi. Bryologist.

[20] Yoshimura I, Yamamoto Y, Nakano T, Finnie J, Kranner I, Beckett R, Varma A (2002). Isolation and culture of lichen photobionts and mycobionts. Protocols in lichenology.

[21] Černajová I, Škaloud P (2020). Lessons from culturing lichen soredia. Symbiosis.

[22] Zakeri Z, Junne S, Neubauer P. Lichens as natural product reservoirs. in: Meyer V, Rapp R. (Hrsg./eds) Mind the Fungi: A publication about the Citizen Science Project *Mind the Fungi.* TU Berlin: University Press. 2020.

[23] Crittenden PD, NewDavid JC, Hawksowrth DL, Campbell FS (1995). Attempted isolation and success in the culturing of a broad spectrum of lichen-forming and lichenicolous fungi. New Phytol.

[24] Sangvichien E, Hawksworth DL, Whalley AJS (2011). Ascospore discharge, germination and culture of fungal partners of tropical lichens, including the use of a novel culture technique. IMA Fungus.

[25] Díaz EM, Zamora JC, Ruibal C, Divakar PK, González-Benítez N, Le Dévéhat F, Chollet M, Ferron S, Sauvager A, Boustie J, Crespo A, Molina MC (2020). Axenic culture and biosynthesis of secondary compounds in lichen symbiotic fungi, the Parmeliaceae. Symbiosis.

[26] Pichler G, Candotto Carniel F, Muggia L, Holzinger A, Tretiach M, Kranner I (2021). Enhanced culturing techniques for the mycobiont isolated from the lichen Xanthoria parietina. Mycol Prog.

[27] Verma N, Behera BC, Upreti D, Divakar P, Shukla V, Bajpai R (2015). In vitro culture of lichen partners: need and implications. Recent advances in lichenology.

[28] Gasulla F, Guéra A, Barreno E (2010). A simple and rapid method for isolating lichen photobionts. Symbiosis.

[29] Armaleo D (1991). Experimental microbiology of lichens: mycelia fragmentation, a novel growth chamber, and the origins of thallus differentiation. Symbiosis..

[30] Muggia L, Grube M (2018). Fungal Diversity in lichens: from extremotolerance to interactions with algae. Life (Basel, Switzerland).

[31] Bando M, Sugino M (1995). Cultivation of the lichen Parmotrema tinctorum in growth cabinets. J Plant Res.

[32] Yoshimura I, Kurokawa T, Yamamoto Y, Kinoshita Y (1993). Development of lichen thalli in vitro. Bryologist.

[33] Delvigne F, Zacchetti B, Fickers P, Fifani B, Roulling F, Lefebvre C, Neubauer P, Junne S (2018). Improving control in microbial cell factories: from single-cell to large-scale bioproduction. FEMS Microbiol Lett.

[34] Vobis G (1977). Studies on the germination of lichen conidia. Lichenologist.

[35] Booth C (1971). Methods in Microbiology.

[36] Lilly VG, Barnett HL (1951). In physiology of fungi.

[37] Deason TR, Bold HC. Phycological studies 1. Exploratory studies of Texas soil algae. University of Texas Publication Nr. 60022. 1960. Doi: 10.1007/978-3-642-56359-1_1

[38] Zakeri Z, Divakar PK, Otte V (2017). Taxonomy and phylogeny of Aspiciliella a resurrected genus of Megasporaceae, including a new species *A. portosantana*. Herzogia..

[39] Zakeri Z, Otte V, Sipman H, Malíček J, Cubas P, Rico VJ, Lenzová V, Svoboda D, Divakar PK (2019). Discovering cryptic species in the *Aspiciliella intermutans* complex (Megasporaceae, Ascomycota)—first results using gene concatenation and coalescent-based species tree approaches. PLoS ONE.

[40] Gardes M, Bruns TD (1993). ITS primers with enhanced specificity for *Basidiomycetes*–application to the identification of mycorrhizae and rusts. Mol Ecol.

[41] White TJ, Bruns TD, Lee S, Taylor J, Innis MA, Gelfand DH, Sninsky JJ, White TJ (1990). Amplification and direct sequencing of fungal ribosomal RNA genes for phylogenetics. PCR protocols: a guide to methods and applications.

[42] Myllys L, Lohtander K, Källersjö M, Tehler A (1999). Sequence insertions and ITS data provide congruent information on Roccella canariensis and R. tuberculata (Arthoniales, Euascomycetes) phylogeny. Mol Phylogenetics Evol..

[43] Lohtander K, Myllys L, Sundin R, Källersjö M, Tehler A (1998). The species pair concept in the lichen Dendrographa leucophaea (Arthoniales): analyses based on ITS sequences. Bryologist.

[44] Kroken S, Taylor JW (2000). Phylogenetic species, reproductive mode, and specificity of the green alga Trebouxia forming lichens with the fungal genus Letharia. Bryologist.

[45] Piercey-Normore MD, DePriest PT (2001). Algal switching among lichen symbioses. Am J Bot.

