# Supplementary material for: Lichen cell factories: methods for the isolation of photobiont and mycobiont partners for defined pure and co-cultivation
Source: Microb Cell Fact. 2022 May 9;21:80. doi: 10.1186/s12934-022-01804-6 (PMC9082883; doi:10.1186/s12934-022-01804-6)
Supplement: Supplementary file 1 — Additional file 1: Table S1. Individual cultures and sample numbers present in the study. [file 12934_2022_1804_MOESM1_ESM.docx]

Table S. 1: Individual cultures and sample numbers present in the study

| **Taxon** | **Locality, Voucher** |
| --- | --- |
| *Cladonia macilenta* | Berlin, Zakeri 0001 |
| *Cladonia fimbriata* | Berlin, Zakeri 0002 |
| *Protoparmeliopsis muralis* | Berlin, Zakeri 0003 |
| *Parmelia sulcata* | Berlin, Zakeri 0004 |
| *Circinaria contorta* | Berlin, Zakeri 0005 |
| *Xanthoria parietina* | Berlin, Zakeri 0006 |
| *Cladonia macilenta* (isolated from Zakeri 0001) | Zakeri F-0001 |
| *Cladonia fimbriata* (isolated from Zakeri 0002) | Zakeri F-0002 |
| *Protoparmeliopsis muralis* (isolated from Zakeri 0003) | Zakeri F-0003 |
| *Parmelia sulcata* (isolated from Zakeri 0004) | Zakeri F-0004 |
| *Circinaria contorta* (isolated from Zakeri 0005) | Zakeri F-0005 |
| *Xanthoria parietina* (isolated from Zakeri 0006) | Zakeri F-0006 |
| *Asterochloris italiana* (isolated from Zakeri 0001) | Zakeri A-0001 |
| *Asterochloris lobophora* (isolated from Zakeri 0002) | Zakeri A-0002 |
| *Coccomyxa sp.* (isolated from Zakeri 0004) | Zakeri A-0004 |
| *Trebouxia sp*. (isolated from Zakeri 0005) | Zakeri A-0005 |
| *Trebouxia decolorans* (isolated from Zakeri 0006) | Zakeri A-0006 |
